# Study on the Viable but Non-culturable (VBNC) State Formation of *Staphylococcus aureus* and Its Control in Food System

**DOI:** 10.3389/fmicb.2020.599739

**Published:** 2020-11-26

**Authors:** Yanmei Li, Teng-Yi Huang, Yuzhu Mao, Yanni Chen, Fan Shi, Ruixin Peng, Jinxuan Chen, Lei Yuan, Caiying Bai, Ling Chen, Kan Wang, Junyan Liu

**Affiliations:** ^1^Department of Haematology, Guangzhou Women and Children’s Medical Center, Guangzhou Medical University, Guangzhou, China; ^2^Department of Laboratory Medicine, The Second Affiliated Hospital of Shantou University Medical College, Shantou, China; ^3^Guangdong Province Key Laboratory for Green Processing of Natural Products and Product Safety, School of Food Science and Engineering, South China University of Technology, Guangzhou, China; ^4^College of Food Science and Engineering, Yangzhou University, Yangzhou, China; ^5^Guangdong Women and Children Hospital, Guangzhou, China; ^6^Research Institute for Food Nutrition and Human Health, Guangzhou, China; ^7^Research Center for Translational Medicine, The Second Affiliated Hospital, Medical College of Shantou University, Shantou, China; ^8^Department of Civil and Environmental Engineering, University of Maryland, College Park, MD, United States

**Keywords:** *Staphylococcus aureus*, VBNC state, induction, control, formation

## Abstract

A Viable but non-culturable (VBNC) state is a bacterial survival strategy under reverse conditions. It poses a significant challenge for public health and food safety. In this study, the effect of external environmental conditions including acid, nutrition, and salt concentrations on the formation of *S. aureus* VBNC states at low temperatures were investigated. Different acidity and nutritional conditions were then applied to food products to control the VBNC state formation. Four different concentration levels of each factor (acid, nutrition, and salt) were selected in a total of 16 experimental groups. Nutrition showed the highest influence on the VBNC state formation *S. aureus*, followed by acid and salt. The addition of 1% acetic acid could directly kill *S. aureus* cells and inhibit the formation of the VBNC state with a nutrition concentration of 25, 50, and 100%. A propidium monoazide-polymerase chain reaction (PMA-PCR) assay was applied and considered as a rapid and sensitive method to detect *S. aureus* in VBNC state with the detection limit of 10^4^ CFU/mL.

## Highlights

- The stress of nutrition, acid and salt could induce the VBNC state formation of *S. aureus* under low temperature.
- The effect of external environmental conditions on the state of VBNC formation of *S. aureus* was: nutrition > acid > salt concentrations.
- Addition of 1% acetic acid could directly kill the *S. aureus* and inhibit VBNC state formation with nutrition concentration of 25, 50, and 100%.
- The PMA-PCR assay is able to be applied on the detection of *S. aureus* VBNC cells with a detection limit at 10^4^ CFU/mL.

## Introduction

Food-borne pathogens can cause diseases by contaminating food products and are the cause of serious concerns in public health and food safety. *Staphylococcus aureus* is widely distributed in the environment including air, water, and the surface of the skin, and has been found in raw meat, milk and dairy products, frozen products, and cooked foods (Xu et al., 2012a,b; Bao et al., 2017a,c). It is the source of major concern in the food industry due to its multi-drug resistance and virulence (Miao et al., 2017c; Jia et al., 2018). Foodborne outbreaks with vomiting cases caused by *S. aureus* have been frequently reported in recent years (Bennett et al., 2013). It can produce Staphylococcus enterotoxins (SEs) including SEA, SEB, SEC, SED, and SEE which can cause severe food poisoning incidents (Xu et al., 1982). Besides, Panton-Valentine leukocidin (PVL) can also cause food poisoning with a high mortality rate (Kraushaar and Fetsch, 2014; Liu et al., 2019).

The “Viable but non-culturable” (VBNC) state, first reported by Xu et al. in 1982, is considered to be a survival strategy of non-spore-forming bacteria in response to adverse conditions (Xu et al., 1982; Oliver, 2010; Liu et al., 2018, 2018a,b). Environmental stresses including low temperature, nutrient-limited conditions, high salt, low pH, and even UV-induced conditions have been reported to induce the formation of a VBNC state (Foster, 1999; Ramaiah et al., 2002; Xu et al., 2008a,b; Cunningham et al., 2009; Wang et al., 2011; Guo et al., 2019). At present, 85 species of bacteria have been confirmed as capable of entering into a VBNC state, including 18 non-pathogenic and 67 pathogenic species (Li et al., 2014; Bao et al., 2017b; Miao et al., 2017a,b , c). VBNC cells are alive with low metabolic activity, and capable of producing biological toxins. *Shigella dysenteriae* type1 retained Shiga toxin encoding gene (*stx*) and produced toxin in the VBNC state (Rahman et al., 1996; Lin et al., 2016). VBNC *Escherichia coli* O157 cells had a higher expression of *rfbE* and relatively lower expression of *stx1* and *stx2* genes compared to normal cells (Liu et al., 2017b; Xu X. et al., 2017; Xu J. et al., 2017; Zhao et al., 2018a,b). Furthermore, VBNC cells can resuscitate when in suitable conditions (Pinto et al., 2015; Xu et al., 2016a,b, c; Miao et al., 2018). Therefore, VBNC pathogens pose a serious threat to food safety and human health.

The traditional detection method for foodborne microbes is culturing-based. However, in the VBNC state, bacteria remain metabolic activity but below detection levels, indicating the ability to cause false negative detection by culturing-based method (Xu M. E. et al., 2011; Xu L. et al., 2011; Xu Z. et al., 2011; Pinto et al., 2015; Miao et al., 2016). Thus, food safety incidents may occur if contaminated by the foodborne pathogen in the VBNC state (Xu et al., 2012a,b; Liu et al., 2017b) and traditional culturing-based methods cannot be trusted to detect VBNC cells. Furthermore, this method cannot identify living and dead cells which is a major limitation in nucleic acid diagnosis (Xu et al., 2010; Zhong et al., 2016; Lin et al., 2017). However, some reagents including photoreactive DNA-binding dyes ethidium bromide monoazide (EMA) and propidium monoazide (PMA) can be used to amplify DNA in dead cells. Nucleic acid amplification methods have been combined with EMA/PMA and widely developed for the detection of pathogenic bacteria in a VBNC state, including PMA-PCR and PMA-LAMP (Li et al., 2017; Xie et al., 2017a,b; Liu et al., 2018; Zhong and Zhao, 2018).

This study aimed to investigate the effect of nutrition, acid, and salt concentrations on the viability and culturability of *S. aureus* at low temperature (4 and −20°C) to obtain a better understanding of the conditions of the formation of the VBNC state in food systems and to enable us to control it. A PMA-PCR assay was applied to detect the VBNC cells of *S. aureus.*

## Materials and Methods

### Bacterial Strain and Culture Conditions

The stain used in this study was *S. aureus* ATCC25923, which was maintained as glycerol stock and stored at −80°C before use. The strain was streaked on tryptic soy agar (TSA) plate and incubated at 37°C for 24 h to recover. A single colony was then inoculated into 2 mL of TSB and incubated at 37°C with 150 rpm for 12 h prior to further experiments.

### VBNC State Induction

The bacterial culture was inoculated into TSB with 1:100 dilution and was incubated until it reached the exponential phase according to the growth curve (data not shown). The exponential phase culture was centrifuged at 5,000 × g for 10 min and the cells were washed with 1 × phosphate buffer solution (PBS). The washed culture was resuspended in induction groups (Table 1) to a final concentration of approximately 10^7^ CFU/mL. To avoid the effects of continuous freeze-thawing, the induction system was separated into multiple 1.5 mL centrifuge tubes. Subsequently, the tubes were placed at 4 and −20°C, respectively, to induce the VBNC state.

**TABLE 1:** The VBNC state induction groups.

Groups	TSB (%)	NaCl (%) (m/v)	Acetic acid (%) (v/v)
1	0	0.9	0
2	25	0.9	0.3
3	50	0.9	0.7
4	100	0.9	1
5	25	10	0
6	0	10	0.3
7	100	10	0.7
8	50	10	1
9	50	20	0
10	100	20	0.3
11	0	20	0.7
12	25	20	1
13	100	30	0
14	50	30	0.3
15	25	30	0.7
16	0	30	1

### Determination of VBNC State

To determine the culturability of *S. aureus* cells, the plate counting method was applied to identify the culturable cell number. The induction culture was serially diluted with 0.9% NaCl and inoculated on TSA followed by incubation at 37°C for 24 h. When culturable, the cell number was < 1 CFU/mL for 3 days, and the cells were considered to be non-culturable (Deng et al., 2015). In addition, the LIVE/DEAD^®^ BacLight^TM^ bacterial viability kit (Thermo Fisher Scientific, China) combined with fluorescence microscopy was used to determine whether the non-culturable cells were in the VBNC state following the manufacturer’s instructions.

### Control of VBNC State

According to VBNC state induction results, suitable concentrations of nutrition, salt, and acid were selected to inhibit the formation of the VBNC state. The bacterial culture was washed and resuspended at the concentration of 5 × 10^7^ CFU/mL with a total volume of 30 mL and stored at 4 and −20°C, respectively. The culturable cell number was measured by plate counting after 3 days (Tables 2, 3).

**TABLE 2:** Inhibition assay of acidity on the VBNC state formation.

Group	TSB (%)	NaCl (%) (m/v)	Acetic acid (%) (v/v)
1	0 25	0.9	0.7
2			1
3	0 25	0.9	1.0
4			1
5	0 25	10	0.7
6			1

**TABLE 3:** Inhibition assay of nutritional status on the VBNC state formation.

Group	TSB (%)	NaCl (%) (m/v)	Acetic acid (%) (v/v)
1	0	0.9	0.3
2			0.7
3			1
4	25	0.9	0.7
5			1.0

### Control of VBNC State in Rice Product

Twenty-five grams of Cantonese rice cake (Guangzhou Restaurant, Guangzhou, China) was added to 225 mL of 0.9% NaCl and determined as a 100% food sample medium. Accordingly, 25 and 50% food sample medium were prepared with sterilization. 2 mL of *S. aureus* culture at exponential growth phase were centrifuged at 4°C and washed with 0.9% NaCl before resuspended with sterilized food sample medium to a final concentration of 5 × 10^7^ CFU/mL as initial induction concentration. Simultaneously, the filtered acetic acid solution was added into the food induction group at a final concentration of 1% (v/v). Then, the final induction group was stored at 4°C for 3 days and the culturability and viability were identified by plate counting method and LIVE/DEAD^®^ BacLight^TM^ bacterial viability kit, respectively.

### PMA-PCR Assay

Twenty-five grams of Cantonese rice cake mixed with 225 mL saline was prepared as a diluting solution. The bacterial culture of *S. aureus* in the VBNC state was diluted to the final concentrations of 10^6^, 10^5^, 10^4^, 10^3^, 10^2^, and 10 cells/mL using the diluting solution, respectively. The PMA reagent was used at the concentration of 5 μg/mL. Subsequently, the detection samples mixed with PMA were incubated in the dark at room temperature for 10 min before the tubes were placed horizontally on ice exposed to a halogen lamp (650 W) at a distance of 15 cm for 15 min to complete the combination of DNA and PMA (Chen et al., 2020). The mixed samples were centrifuged at 10,000 rpm for 5 min. DNA from the precipitated cells was isolated using a DNA extraction kit (Dongsheng Biotech, Guangzhou) following the manufacturer’s instruction.

The PCR assay was performed at a total volume of 25 μL. The reaction system consists of 12.5 μL 2 × Taq PCR MasterMix (Dongsheng Biotech, Guangzhou), 3 μM each of forward and reverse primers (*femA*-F: AGGTATAGACTTCGATG TTTCAAATCGCGGTCCAGTG; *femA*-R: TTGTAGCTTCAGATATGGAAACCAA TCATTAC CAGCA), 2 μL of DNA template and added up to 25 μL with nuclease-free water. A mixture with 2 μL of nuclease-free water was used (instead of DNA) as a negative control. The protocol of PCR assay was as followed: 5 min denaturation at 95°C, 32 cycles of amplification at 95°C for 30 s, 52°C 30 s, 72°C for 35 s and final inactivation at 72°C for 5 min. The PCR products were detected by electrophoresis on 1.5% agarose gels and observed under UV light.

## Results and Discussion

### Culturability of *S. aureus* During Induction

**FIGURE 1:**
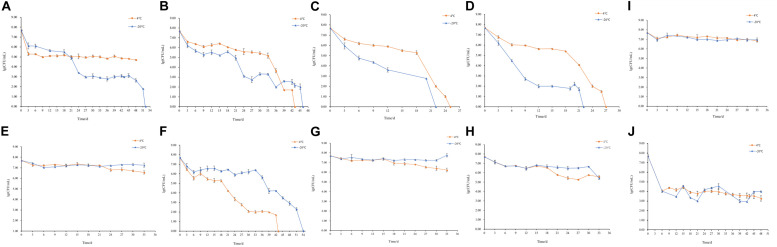
Culturable cell numbers of *S. aureus* under different conditions stored at 4 and –20°C (**A**–**J,** represent the cell culturability of *S. aureus* in induction groups 1, 2, 3, 4, 5, 7, 9, 10, 13, and 14, respectively).

The changes of culturable cell numbers during VBNC state induction are shown in Figure 1. In induction group 1, for which stored at −20°C, the culturable cell number decreased to 0 in 52 days, while at 4°C the culturable cell number dropped in the first 3 days and remained unchanged (Figure 1A). In induction group 2, the culturable number declined to 0 in 43 days (−20°C) and 46 days (4°C), respectively (Figure 1B), which was similar to the result of induction groups 3 and 4 but with the longer induction time (Figures 1C,D). As for induction group 7, the culturable cell number reduced to 0 after stored at 4 and −20°C for 43 days and 54 days, respectively (Figure 1F). And in induction groups 5, 9, 10, 13, and 14, the culturable cell numbers remain the same which indicated the cells were unable to enter into the VBNC state (Figures 1E,G–J). The cells in induction groups 6, 8, 11, 12, 15, and 16 were non-culturable in 3 days. The total times for *S. aureus* cells to become non-culturable are listed in Table 4.

**TABLE 4:** Duration for culturable cell number decreased to 0.

Group	4°C	−20°C	Group	4°C	−20°C
1	+	52 days	9	+	+
2	46 days	43 days	10	+	+
3	25 days	22 days	11	/	/
4	27 days	22 days	12	/	/
5	+	+	13	+	+
6	/	/	14	+	+
7	43 days	54 days	15	/	/
8	/	/	16	/	/

*“ + ” stands for S. aureus is culturable and “/” stands for the number of culturable S. aureus in 3 d dropped to 0.*

### Viability of *S. aureus* During Induction

**FIGURE 2:**
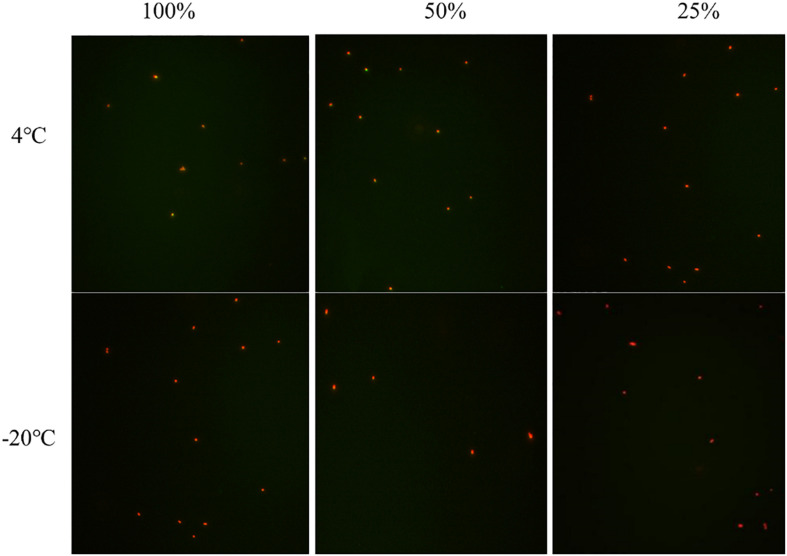
The viability of nonculturable *S. aureus* stored at different conditions with fluorescence microscope.

The viability of *S. aureus* during the induction was observed by the fluorescence microscope after the treatment of the LIVE/DEAD^®^ BacLight^TM^ bacterial viability kit. In induction group 1 at −20°C, when the culturable cell number decreased to 0, viable cells still existed indicating that *S. aureus* can enter into VBNC state in saline at −20°C (Figure 2). The same results were obtained in induction group 2 at 4 and −20°C (Figure 2). As for induction groups 3, 4, and 7 at 4°C, a small percentage of cells entered into VBNC state (Figure 2). However, most cells were dead at −20°C (Figure 2).

In summary, under low temperature (4°C) and strong acidity with sufficient nutrition (medium concentration ≥ 50%), *S. aureus* could enter into VBNC state within a short time. Similar results were obtained under low salt and weak acidic environment with insufficient nutrition but with longer induction time, as well as the in conditions that were oligotrophic and acid-free. Briefly, under reverse conditions, including insufficient nutrition with weak acid and sufficient nutrition with a strong acid, it was easier for *S. aureus* to enter into VBNC state at 4°C but more difficult to survive in freezing conditions (−20°C). These results showed that the key conditions for the VBNC state formation of *S. aureus* were adequate nutrition with strong acid at 4°C, insufficient nutrition with weak acid at 4°C, and oligotrophic system at −20°C.

*S. aureus* was able to enter into VBNC state under strong acid with adequate nutrition but not under strong acid that lacked nutrition, indicating with the treatment of strong acid, nutrition plays an important role during the formation of VBNC state. By comparing induction groups 1 and 2, under weak acid, it took cells in group 2 a shorter time to enter into VBNC state, demonstrating that weak acid may have an active contribution to the formation of VBNC state. Due to the salt-tolerant property of *S. aureus*, salt concentration had no significant effect. Under insufficient nutrition (medium concentration ≤ 50%) with strong acid [concentration of acetic acid (v/v) ≥ 0.7%] and high salt concentration (≥10%) without nutrition, the cells died within 3 days. Therefore, nutrition had the strongest effect on the formation of the VBNC state, followed by the concentration of acetic acid and salt.

*S. aureus* cells with higher ATP concentration would enter into the VBNC state instead of dying. Similar phenomenon has been found in the VBNC *L. monocytogenes* (Lindbäck et al., 2010; Bai et al., 2019). ATP synthase was also found upregulated in VBNC *Vibrio parahaemolyticus* cells (Lai et al., 2009). The upregulation of genes or proteins related to ATP accumulation offset ATP consumption in VBNC bacteria might be due to the survival mechanism of VBNC *S. aureus* under the reverse condition and need to be confirmed by further study on the expression of ATP-related genes or proteins in VBNC *S. aureus* cells (Bai et al., 2019). In the VBNC *S. aureus* cells, the mutational inactivation of catalase (KatA) or superoxide dismutase (SodA) encoded by *katA* and *sodA* gene was present. The changes on the expression of genes rendered cell hypersensitive to seawater with a high concentration of salt at 4°C (Masmoudi et al., 2010).

### Control of VBNC State

Under weak acid with insufficient nutrition (medium concentration ≤ 25%) conditions, *S. aureus* was capable of entering into VBNC state and acid concentration influenced the survival of *S. aureus* cells. Thus, a further experiment on the inhibitory effect of 0.3, 0.7, and 1.0% acetic acid on the control of VBNC state was performed.

In saline with 0.3, 0.7, and 1.0% acetic acid (induction groups 1, 2, and 3), *S. aureus* lost culturability and viability in 3 days, while low salt concentration with nutrition (medium concentration ≤ 25%) and 0.7%, 1.0% acetic acid (induction group 4 and 5), *S. aureus* remained culturability when stored at 4°C for 3 days. Among all, only the cells in induction group 5 stored at −20°C were non-culturable, indicating that eliminating VBNC state formation only by acetic acid treatment is not sufficient. *S. aureus* died at low nutrition, high salt, and strong acidity, indicating salt concentration can restrain the formation of the VBNC state However, given the low salt concentration in the food processing and storage of rice product, only acid treatment is less effective in eliminating *S. aureus* and its VBNC state (Table 5).

**TABLE 5:** Inhibition of acidity on the formation of VBNC state of *S. aureus.*

Group	Culturability		Viability	
	4°C	−20°C	4°C	−20°C
1	/	/	*–*	*–*
2	/	/	*–*	*–*
3	/	/	*–*	*–*
4	+	+	ND	ND
5	+	+	ND	ND

*“ + ” stands for culturable, “/” for unculturable, “–” for inactive and “ND” for activity is not detected.*

Since *S. aureus* could enter into VBNC state with strong acid treatment, the elimination of VBNC state by different nutrition conditions with strong acid were studied. Under low temperature (4 and −20°C), all cells were dead within 3 days in groups with no nutrition and strong acid (induction groups 1, 3, and 5). *S. aureus* may enter into VBNC under the treatment of some nutrition and low salt with strong acid. These results indicated that in low salt and strong acid environment, the VBNC state of *S. aureus* cannot be eliminated by only reducing nutrients. Thus, the control of VBNC state formation can be achieved by changing nutrition concentration in combination with other treatments. One way of eliminating the VBNC state of *S. aureus*, which could be applicable in food processing or used when cleaning equipment, would be to provide a condition of no nutrition with acid (Table 6).

**TABLE 6:** Inhibition of nutritional status on the VBNC state formation.

Group	Culturability		Viability	
	4°C	−20°C	4°C	−20°C
1	−	−	−	−
2	+	+	ND	ND
3	−	−	−	−
4	+	−	ND	0
5	−	−	−	−
6	+	+	ND	ND

*“ + ” stands for culturable, “/” for unculturable, “−” for inactive and “ND” for activity is not detected.*

Recently, several studies have reported on the formation of Staphylococcus biofilm on different surfaces during food processing, including polystyrene, polypropylene, stainless steel, and glass (Sattar et al., 2001; DeVita et al., 2007; Simon and Sanjeev, 2007). VBNC state induced in *S. aureus* biofilm under the antibiotic pressure has also been confirmed by RT-PCR (Pasquaroli et al., 2013, 2014). *S. aureus*, as well as its VBNC state formation, is emerging as a major concern of food product contamination and poses a threat to human health.

### Control of VBNC State in Rice Product

**FIGURE 3:**
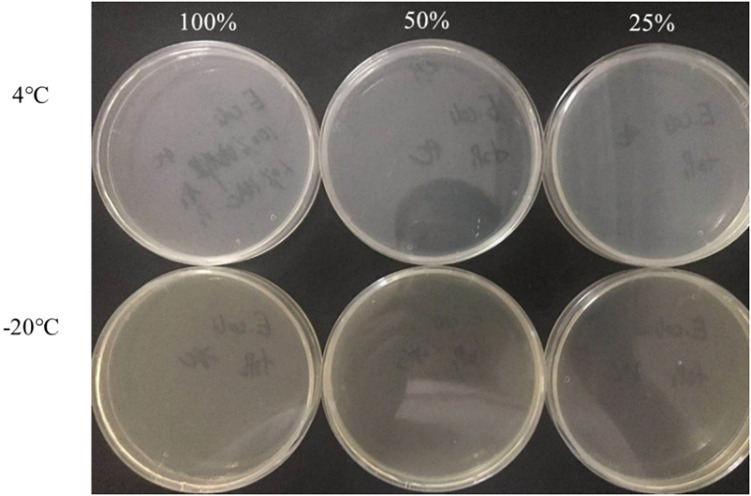
The culturable cell number of *S. aureus* inoculated in the 1.0% (v/v) acetic acid containing 100, 50, 25% nutrients at low temperature for 3 days.

**FIGURE 4:**
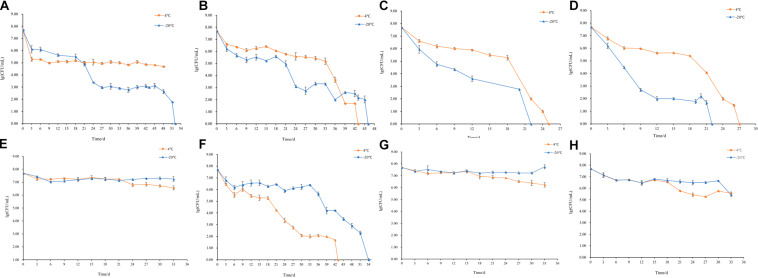
The viability of non-culturable *S. aureus* stored at different conditions **(A)** group 1 (–20°C); **(B,C)** group 2 (4°C, –20°C); **(D,E)** group 3 (4°C, –20°C); **(F,G)** group 4 (4°C, 20°C); **(H)** group 7 (4°C).

The culturable cell numbers under low temperature stress are shown in Figure 3. In food systems with 100, 50, and 25% nutrients and 1.0% acetic acid, the cells lost their culturability in 3 days. Observation under a fluorescence microscope (Figure 4) confirmed that all cells were dead, which is different from the results in the induction in TSB due to complex food matrix with unfavorable factors. Thus, 1% of acetic acid can be applied in the control of normal and VBNC state *S. aureus* cells in rice products.

Foodborne pathogens and spoilage bacteria in the VBNC state can produce toxins and cause food spoilage. This is emerging as a leading concern for the food industry (Xu et al., 2009; Liu et al., 2017a). Over the past few decades, it has been confirmed that VBNC cells are capable of recovering, with restored metabolic activity. However, resuscitation conditions vary among species and strains. VBNC *E. coli* O157:H7 and *Vibrio vulnificus* cells can recover with the treatment of fresh TSB and a temperature upshift (Dinu and Bach, 2013; Zhao et al., 2013; Zhong et al., 2013; Rao et al., 2014; Xu et al., 2018). Moreover, the VBNC state pathogens recover or maintain virulence after resuscitation (Cappelier et al., 2007; You et al., 2012; Zeng et al., 2013). Several foodborne outbreaks were due to the resuscitation of VBNC cells (Xu et al., 2007; Zhao et al., 2017; Wen et al., 2020). Therefore, the control of the VBNC state formation in food products is of importance.

### Application of PMA-PCR on VBNC Cell Detection in Rice Product

The detection limit of PMA-PCR for detection of the VBNC state *S. aureus* in rice products was 10^4^ CFU/mL. Compared with the conventional culturing-based method, which uses the LIVE/DEAD^®^ BacLight^TM^ fluorescence staining method to detect VBNC cells, the PMA-PCR assay can detect specific concentrations of VBNC cells with high rapidity and sensitivity (Yoon et al., 2019).

## Conclusion

This study investigated the impact of three elements including nutrition, acid and salt concentrations in food systems on the VBNC state formation of *S. aureus*. Nutrition showed the highest influence on the VBNC state formation *S. aureus*, followed by acid and salt. The addition of 1% acetic acid could directly kill *S. aureus* cells and inhibit the formation of VBNC states with a nutrition concentration of 25, 50, and 100%. Propidium monoazide-polymerase chain reaction (PMA-PCR) assay was applied and considered to be a rapid and sensitive method for detecting *S. aureus* in the VBNC state, with the detection limit of 10^4^ CFU/mL.

## Data Availability Statement

The original contributions presented in the study are included in the article/supplementary material, further inquiries can be directed to the corresponding authors.

## Author Contributions

JL and KW conceived of the study and participated in its design and coordination. T-YH, YM, YC, FS, RP, and JC performed the experimental work. CB, LY, and LC analyzed the data. JL prepared and revised this manuscript. All authors reviewed and approved the final manuscript.

## Conflict of Interest

The authors declare that the research was conducted in the absence of any commercial or financial relationships that could be construed as a potential conflict of interest.
